# Pairwise analysis of plasma cell-free DNA before and after palliative second-line paclitaxel plus ramucirumab treatment in patients with metastatic gastric cancer

**DOI:** 10.1007/s10120-025-01604-y

**Published:** 2025-03-27

**Authors:** Ji-Won Kim, Dong Soo Kyung, Won Yeong Ko, Hwang-Phill Kim, Sung-Hyun Hwang, Kui-Jin Kim, Ju Hyun Lee, Jeongmin Seo, Minsu Kang, Eun Hee Jung, Koung Jin Suh, Se Hyun Kim, Jin Won Kim, Yu Jung Kim, Jee Hyun Kim, Keun-Wook Lee

**Affiliations:** 1 Department of Internal Medicine, Seoul National University Bundang Hospital, Seoul National University College of Medicine, 82 Gumi-Ro-173-Beon-Gil, Bundang-Gu, Seongnam, 13620 Republic of Korea; 2 IMBdx, Inc., 131 Gasandigital-1-Ro, Geumcheon-Gu, Seoul, 08506 Republic of Korea; 3 Biomedical Research Institute, Seoul National University Bundang Hospital, Seoul National University College of Medicine, 82 Gumi-Ro-173-Beon-Gil, Bundang-Gu, Seongnam, 13620 Republic of Korea; 4 Department of Statistics, Hankuk University of Foreign Studies, 81 Oedae-Ro, Cheoin-Gu, Yongin, 17035 Republic of Korea

**Keywords:** Cell-free DNA, Circulating tumor DNA, Gastric cancer, Anti-cancer therapy, Clonal evolution

## Abstract

**Background:**

This study compared plasma cell-free DNA (cfDNA) and tumor tissue DNA (ttDNA) to explore the clinical applicability of cfDNA in patients with metastatic gastric cancer (mGC) receiving palliative second-line paclitaxel + ramucirumab treatment.

**Methods:**

Targeted sequencing of 106 genes was conducted using germline DNA and cfDNA at baseline (baseline-cfDNA) and progressive disease (PD-cfDNA). The results were compared with those of ttDNA-based cancer panel data.

**Results:**

Of 76 consecutive patients, 46 (27 males; median age 57.5 [range, 32–73] years) who had all three samples were included. Combined analysis of ttDNA and baseline-cfDNA revealed that *TP53* (58.7%) was the most frequently mutated gene, followed by *CDH1* (26.1%), *KRAS* (21.7%), and *APC* (13.0%). For these genes, the sensitivity and positive predictive value of baseline-cfDNA over ttDNA were 71.8% and 51.9%, respectively. When baseline-cfDNA and PD-cfDNA results were combined, 34 patients (73.9%) were found to have additional mutations compared with ttDNA results alone. PD-cfDNA analysis revealed 14 novel pathogenic mutations in ten patients (21.7%). At baseline, patients with a high circulating tumor DNA fraction concentration showed a significantly shorter progression-free survival (PFS) (P = 0.016) in univariable and multivariable analyses. High maximal variant allele frequency (VAF) (P = 0.022), high sum of VAF (P = 0.028), and high *TP53* VAF (P = 0.022) were associated with worse PFS in univariable analysis.

**Conclusions:**

Although cfDNA alone cannot replace ttDNA entirely, cfDNA analysis revealed additional mutations. Notably, PD-cfDNA analysis revealed novel pathogenic mutations that emerged during treatment. Moreover, the baseline circulating tumor DNA fraction concentration and VAF values were associated with longer PFS.

**Supplementary Information:**

The online version contains supplementary material available at 10.1007/s10120-025-01604-y.

## Introduction

Gastric cancer (GC) is the sixth most common cancer and the third most common cause of cancer-related death worldwide [1]. In the Republic of Korea, it is the fourth most common cancer and the fourth most common cause of cancer-related death [2]. A significant number of patients with GC are diagnosed with distant metastasis and have an unfavorable prognosis despite recent advancements in palliative anti-cancer therapy. The poor prognosis of these patients is attributed to the clonal evolution of cancer cells, which results in acquired resistance to systemic agents [3]. Therefore, longitudinal genomic analysis of cancer samples, both at baseline and progressive disease (PD), has become increasingly important for understanding the resistance mechanisms of systemic agents.

The combination of ramucirumab, an anti-*VEGFR2* monoclonal antibody, with paclitaxel significantly increased the overall survival (OS) of patients with metastatic GC (mGC) in whom palliative first-line treatment had failed [4]. However, the median progression-free survival (PFS) of this regimen was merely 4.4 months, and most patients eventually experienced disease progression. Therefore, the mechanisms underlying the resistance to these drugs need to be elucidated. Moreover, validated predictive biomarkers for anti-angiogenic agents remain to be developed.

Next-generation sequencing (NGS) has allowed us to comprehensively understand the genomic alterations in GC [5, 6]. In addition to cancer panel sequencing using tumor tissue DNA (ttDNA), plasma cell-free DNA (cfDNA) analysis has recently emerged as an appealing alternative in clinical oncology [7]. This study aimed to determine how accurately the genetic landscape revealed by cfDNA sequencing mirrored the results of ttDNA sequencing. Furthermore, the clinical applicability of cfDNA was explored to better understand clonal evolution during anti-cancer therapy.

## Materials and methods

### GC patient cohort

Patients were recruited from Seoul National University Bundang Hospital (SNUBH), Republic of Korea. DNA samples were collected from each patient from four different sources: ttDNA from tumor tissue, genomic DNA (gDNA) from blood mononuclear cells, cfDNA at baseline (baseline-cfDNA), and cfDNA at PD (PD-cfDNA) established based on the Response Evaluation Criteria in Solid Tumor (RECIST) version 1.1 [8]. This study included patients for whom ttDNA analysis data were available. For ttDNA analysis, four different cancer panels were used: Theragen, SNUBH Pan-Cancer Panel version 1 (SNUBH_V1), SNUBH Pan-Cancer Panel version 2 (SNUBH_V2), and Illumina TruSight Oncology 500 (TS500). The Theragen panel is a research-purpose cancer panel specifically designed for GC (Theragen Bio, Seongnam, Republic of Korea), whereas the other three panels are used in routine clinical practice.

All participants provided written informed consent. This study was conducted in compliance with the Declaration of Helsinki and was approved by an institutional review board (B-1809/492–005).

### Blood sample collection and cfDNA extraction

Plasma isolation was consistently performed within 30 min of blood collection to prevent the degradation of cfDNA and release of gDNA from blood cell lysis. Upon collection, each blood sample was centrifuged with Ficoll-Paque Plus (Cytiva, Marlborough, MA, USA) at 2,000 rpm for 15 min without interruption. The resulting plasma was scrutinized before further processing to ensure quality. To eliminate cell debris, an additional centrifugation step was performed at 16,000 × g under 4 °C for 15 min. Following this, 1 mL aliquots of the plasma were stored in new 1.5 mL microcentrifuge tubes at -80 °C until extraction. cfDNA was isolated from plasma using the QIAamp Circulating Nucleic Acid Kit (Qiagen, Valencia, CA, USA). cfDNA was quantified using a 4200 TapeStation (Agilent Technologies, Santa Clara, CA, USA). Peripheral blood mononuclear cells (PBMCs) were isolated according to an established protocol, and gDNA from PBMCs was isolated using the QIAamp DNA Mini Kit (Qiagen, USA). All experimental procedures adhered to the guidelines for pre-analytical conditions for cfDNA analysis [9–11].

### Targeted panel sequencing

For targeted panel sequencing, DNA NGS libraries were constructed using the IMBdx NGS DNA Library Prep Kit. Solution-based target enrichment was conducted at IMBdx, Inc. (Seoul, Republic of Korea), using the AlphaLiquid®100 (AL100) target capture panel. This panel encompassed 106 cancer-related genes and was designed to cover the entire exons of these genes (Supplementary Table 1). The captured DNA libraries were sequenced on the Illumina NextSeq 550 platform (Illumina, San Diego, CA, USA) in the 150-bp paired-end mode. All sequencing reads were initially generated in a bcl format and then de-multiplexed into fastq files. The fastq files were then trimmed to remove adaptor sequences and aligned with the human reference genome, hg38, using the BWA-MEM algorithm. The reads mapped to the AL100 target regions were extracted. Sequences aligned to the AL100 target regions were then isolated and condensed using Gencore [12].

### Identification of somatic mutations from cfDNA

Initial single-nucleotide variant (SNV) and small insertion or deletion (indel) calls were compiled based on the UniqSeq protocol, which processes multiple tubes independently. Subsequently, a series of in-house IMBdx filtering steps were applied. These calls were scored using a machine learning model to distinguish true from false variants and were further annotated for functional effect prediction [13].

To detect copy number variations (CNVs), a reference sequencing depth profile was prebuilt for the exon regions targeted by AL100 using 50 normal samples [14]. For each gene, CNV detection was performed to assess whether the normalized depth profile of the tested sample was significantly higher than that of the reference profile.

Analytical sensitivity and specificity were evaluated using Seraseq® ctDNA Mutation Mix v2 and Complete™ Mutation Mix reference materials, which contained known SNVs, small indels, and CNVs. SNVs were examined at 24 markers, and small indels were assessed at 13 markers using multiple replicates, covering two independent locations over three days with three replicates per run. For CNVs, reference materials with a more diverse spectrum of allele frequencies were tested for *ERBB2*, *MET*, and *MYC* [13].

Next, germline mutations considered clonal hematopoiesis of indeterminate potential (CHIP) were removed by referencing alterations from the gDNA samples. A cut-off for cfDNA mutations was applied, requiring a variant allele frequency (VAF) of ≥ 0.003 and an altered allele count of ≥ 6 to filter out noise, contamination, or sequencing errors. Any unexpected false positives were curated by visual inspection of longitudinal cfDNA mutation profiles. For CNVs, amplifications were defined as having a copy number (CN) ≥ 6.

We developed a stream plot to illustrate the changes in the VAF and CN values of variants over time, tracing from ttDNA, through baseline-cfDNA, to PD-cfDNA. The Y-scale of the plot was tailored for each patient and anchored to the maximum VAF or CN values recorded across three samples. The maximal VAF and CN values for each sample were indicated below the X-axis. Meanwhile, SNVs and indels that were not detected were represented by a VAF of 0%. Undetected CNVs in the ttDNA were plotted as copy-neutral (CN = 2).

### Calculation of the circulating tumor DNA (ctDNA) fraction concentration

According to previous literature [15], a 110–160 bp span of cfDNA was defined as a ctDNA fraction. The Pysam package version 0.22.0 was used to estimate the fragment length. Fragments ranging from 110–160 bp in size were divided by the total read counts and multiplied by the cfDNA concentration within the 50–700 bp range.

### Statistical analysis for clinical data

Treatment response was evaluated using RECIST version 1.1 [8]. PFS was calculated from the start of treatment until PD or death from any cause. OS was defined as the time from the start of treatment to death from any cause. Survival outcomes were analyzed using the Kaplan–Meier method and compared using the log-rank test. Multivariable analyses were conducted using the Cox proportional hazard model. The cut-off points of the VAF values were determined to predict the 6-month PFS using the receiver operating characteristic (ROC) analysis. Two-sided P-values less than 0.05 were considered statistically significant. The clinical data were analyzed using IBM SPSS Statistics version 26 (Armonk, NY, USA).

## Results

### Patient characteristics and clinical outcomes

Between January 2019 and June 2021, 76 patients with mGC who received palliative second-line therapy with paclitaxel and ramucirumab were consecutively enrolled. Among them, 46 patients with available tumor NGS results using ttDNA as well as baseline- and PD-cfDNA samples were analyzed (Table 1). Except for one patient, GC062, 45 tumor NGS results from ttDNA were obtained before treatment initiation. The median age was 57.5 (range, 32–73) years. The male-to-female ratio was 27:19. In 34 patients (73.9%) with measurable lesions based on RECIST version 1.1, the objective response rate of paclitaxel plus ramucirumab treatment was 23.5% (Supplementary Table 2). The median follow-up time was 41.2 months using the reverse Kaplan–Meier method. The median PFS and OS were 4.1 (95% confidence interval [CI], 2.7–5.5) months (Supplementary Fig. 1A) and 8.6 (95% CI, 6.7–10.5) months (Supplementary Fig. 1B), respectively.

**Table 1:** Baseline characteristics

Clinical characteristics		Values
Median age (year), median (range)		57.5 (32–73)
Sex, *n* (%)	Male Female	27 (58.7) 19 (41.3)
ECOG PS, *n* (%)	0–1 2	45 (97.8) 1 (2.2)
Site of metastasis, *n* (%)	Peritoneum Lymph node Liver Pleura Bone Ovary Lung Adrenal gland	32 (69.6) 27 (58.7) 16 (34.8) 11 (23.9) 9 (19.6) 6 (13.0) 4 (8.7) 1 (2.2)
Histologic type*, *n* (%)	Tubular adenocarcinoma Mucinous adenocarcinoma Poorly cohesive carcinoma Mixed carcinoma Unknown	21 (45.7) 2 (4.3) 16 (34.8) 4 (8.7) 3 (6.5)
Differentiation, *n* (%)	Well differentiated Moderately differentiated Poorly differentiated Unknown	1 (2.2) 13 (28.3) 18 (39.1) 14 (30.4)
Lauren classification, *n* (%)	Intestinal Diffuse Mixed Unknown	10 (21.7) 22 (47.8) 2 (4.3) 12 (26.1)
*HER2*, *n* (%)	Positive Negative	6 (13.0) 40 (87.0)
MMR, *n* (%)	Proficient Deficient Not done	44 (95.7) 0 (0) 2 (4.3)
EBV in situ hybridization, *n* (%)	Positive Negative Unknown	2 (4.3) 40 (87.0) 4 (8.7)
Disease status at palliative treatment, *n* (%)	Recurrent Initially metastatic	14 (30.4) 32 (69.6)
Measurable lesion by RECIST 1.1, *n* (%)	Measurable Non-measurable	34 (73.9) 12 (26.1)
First-line treatment regimens, *n* (%)	FP FP + Trastuzumab FP + Etoposide Clinical trials with FP backbone	33 (71.7) 6 (13.0) 1 (2.2) 6 (13.0)

*ECOG PS* Eastern Cooperative Oncology Group performance status, *HER2* human epidermal growth factor receptor 2, *MMR* mismatch repair, *EBV* Epstein-Barr virus, *RECIST* Response Evaluation Criteria in Solid Tumors, *FP* fluoropyrimidine + platinum

*Histological type according to the WHO 2010 classification

### Summary of identified somatic mutations

AL100, which covers 106 cancer-related genes, was employed for baseline- and PD-cfDNA analyses. For ttDNA analysis, four clinical NGS panels were used: Theragen (N = 28), SNUBH_V1 (N = 3), SNUBH_V2 (N = 9), and TS500 (N = 6). These panels targeted 286, 99, 559, and 523 cancer-related genes, respectively (Supplementary Table 1). Of the 106 genes in the AL100 panel, 20, 38, 2, and 2 genes were not covered by the Theragen, SNUBH_V1, SNUBH_V2, and TS500 panels, respectively, and the number of genes uniformly covered by all five panels was 61.

After excluding duplicate mutations, 179 mutations were identified in 52 genes (Supplementary Fig. 2). We limited our further analyses to these mutations. These mutations were classified as missense (N = 113), nonsense (N = 19), frameshift (N = 21), in-frame (N = 9), splice site (N = 7), or CN gain (N = 10). PD-cfDNA (N = 130) had the highest occurrence of mutations, followed by baseline-cfDNA (N = 126) and ttDNA (N = 69). Overall, cfDNA yielded a better performance for detecting mutations than did ttDNA; no mutations were found in baseline-cfDNA from 21.7% (10 of 46) of patients, while this percentage was 30.4% (14 of 46) in ttDNA. In 4 patients, no mutations were detected in any of the samples.

### Comparison of the mutational landscape between cfDNA and ttDNA

**Fig. 1:**
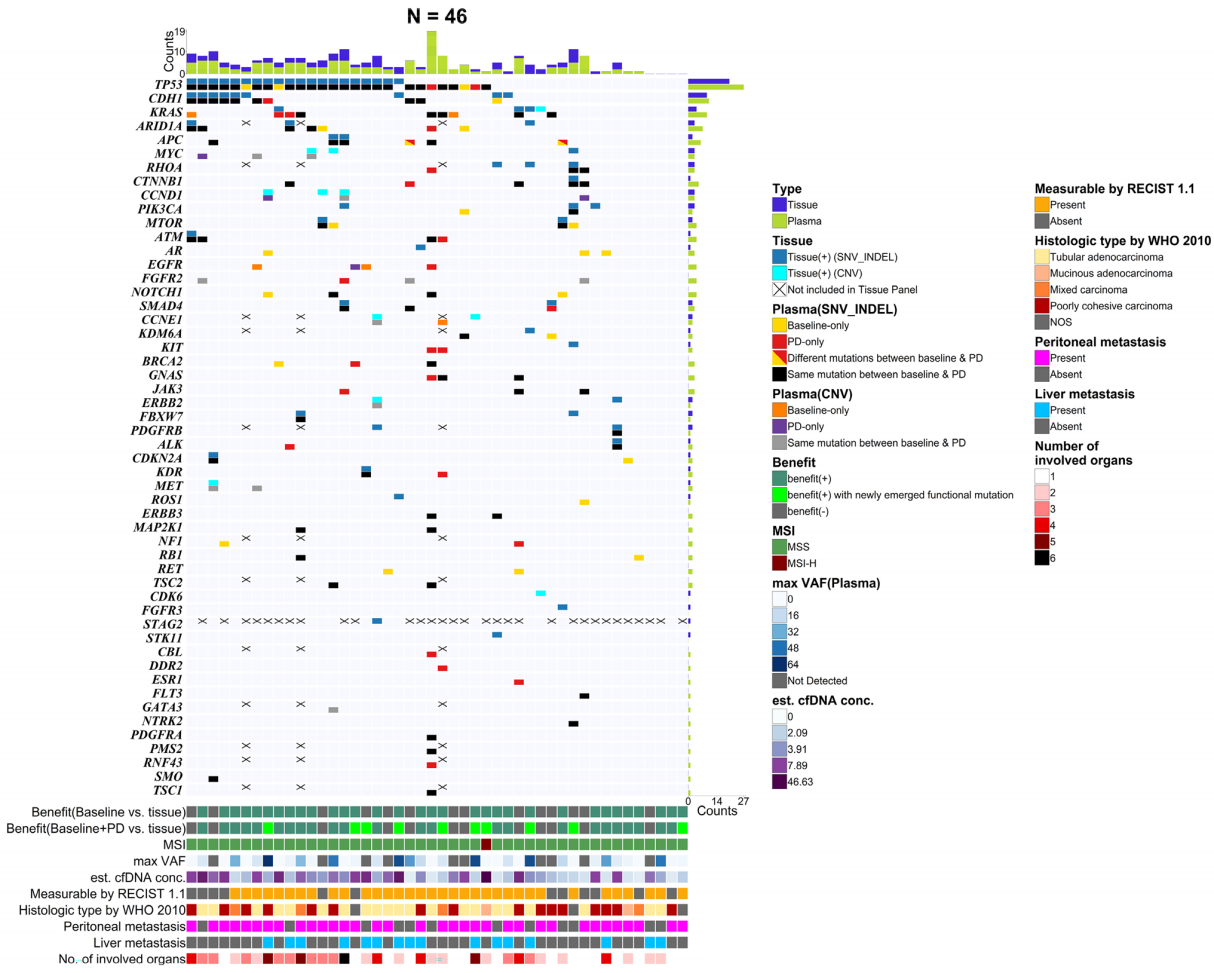
Oncoplot of 46 patients with gastric cancer. For each gene, each patient had two boxes denoting different sample types: ttDNA (upper box) and cfDNA at baseline and PD (lower box). Vertical bar plots at the top and right show the number of mutations with different colors representing different sample types. *SNV* single nucleotide variant, *CNV* copy number variation, *PD* progressive disease, *MSI* microsatellite instability, *MSS* microsatellite stable, *MSI-H* microsatellite instability-high, *VAF* variant allele frequency, *cfDNA* cell-free DNA, *ttDNA* tumor tissue DNA

Combined analysis of ttDNA and baseline-cfDNA revealed that *TP53* (N = 27, 58.7%) was the most frequently mutated gene, followed by *CDH1* (N = 12, 26.1%), *KRAS* (N = 10, 21.7%), and *APC* (N = 6, 13.0%) (Fig. 1). For the top four genes, the sensitivity and positive predictive value (PPV) of baseline-cfDNA compared with those of ttDNA were 71.8% and 51.9%, respectively. When compared with the combined results of ttDNA and baseline-cfDNA, additional PD-cfDNA analysis revealed 33 novel mutations in 24 genes from 15 patients (32.6%).

Next, to evaluate the additional diagnostic performance of cfDNA, we calculated the proportion of patients who were found to harbor additional mutations as determined using cfDNA analysis compared to that with conventional ttDNA analysis. Compared to those based on ttDNA alone, 32 patients (70.0%) were found to have additional mutations in baseline-cfDNA. When combined with baseline-cfDNA and PD-cfDNA, the number of these patients increased to 34 (73.9%) compared with those based on ttDNA alone.

As both baseline- and PD-cfDNA analyses detected more mutations than those in the ttDNA results, we calculated the sensitivity and PPV when baseline-cfDNA results were set as the gold standard to clarify the variant detection performance of the four ttDNA-based cancer panels. Theragen and SNUBH_V1 panels showed similar sensitivities and PPV: 18.4% and 57.1% in Theragen and 15.4% and 50.0% in SNUBH_V1. Compared to these, SNUBH_V2 and TS500 had higher sensitivity and PPV: 76.9% and 71.4% in SNUBH_V2 and 60.0% and 92.3% in TS500.

Thirty-seven genes harbored more than one mutation. The remaining 15 genes had only one mutation. Of these 15 genes, 11 mutations were detected only in cfDNA, seven of which (63.6%) were concordant between baseline-cfDNA and PD-cfDNA, whereas 4 mutations were identified only in ttDNA. Moreover, the following 12 genes had multiple mutations only detected in cfDNA: *EGFR*, *FGFR2*, *NOTCH1*, *BRCA2*, *GNAS*, *JAK3*, *ERBB3*, *MAP2K1*, *NF1*, *RB1*, *RET*, and *TSC2*.

### Individual variation in cancer evolution dynamics

**Fig. 2:**
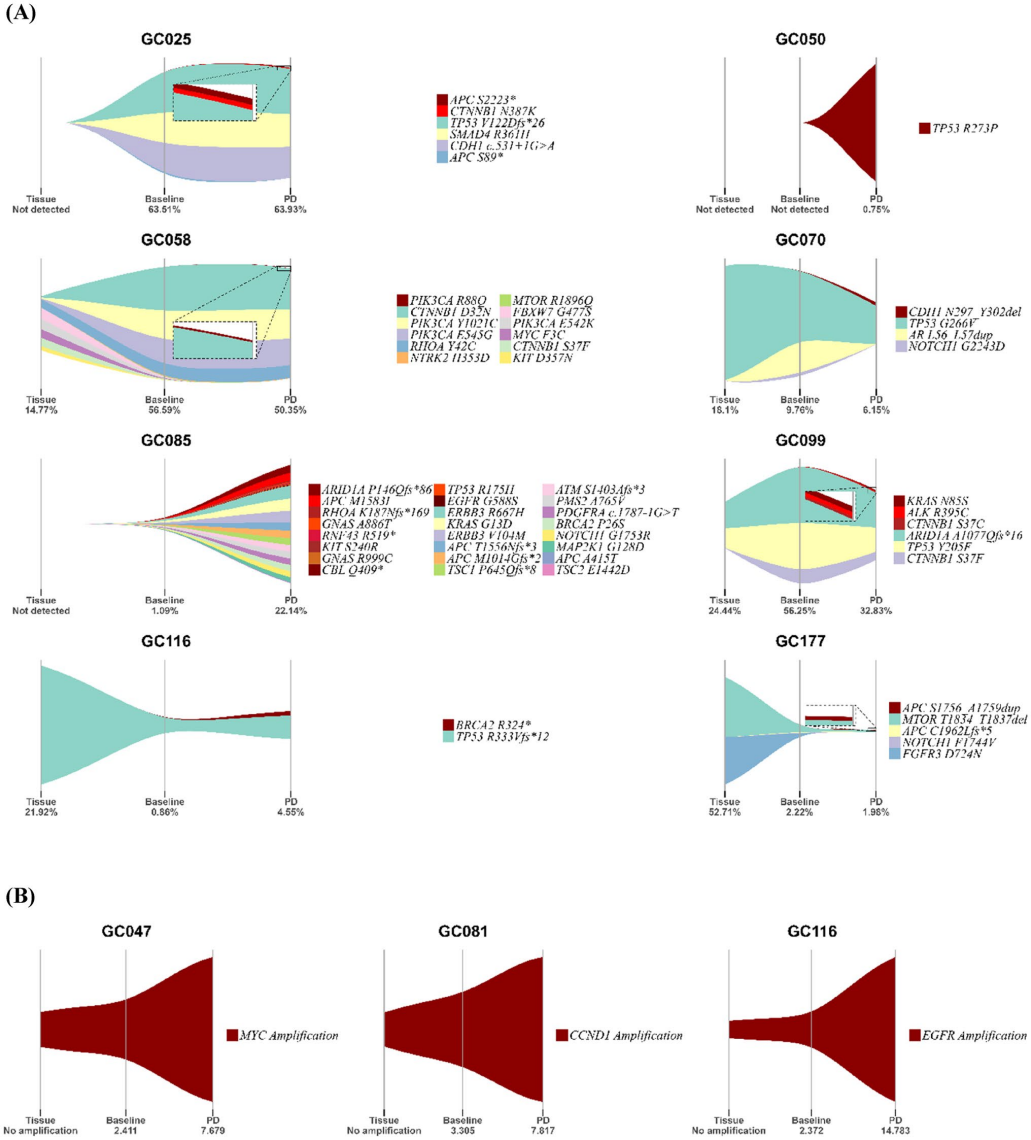
Clonal evolution dynamics before and after the treatment. **A** Variant allele frequency values of ttDNA, baseline-cfDNA, and PD-cfDNA were visualized in eight patients harboring single nucleotide variants and small indels: GC025, GC050, GC058, GC070, GC085, GC099, GC116, and GC177. **B** Copy number values of ttDNA, baseline-cfDNA, and PD-cfDNA were visualized in three patients harboring copy number variations: GC047, GC081, and GC116. *PD* progressive disease, *ttDNA* tumor tissue DNA, *cfDNA* cell-free DNA

We focused on newly emerged somatic mutations in the PD-cfDNA samples because these mutations could implicate clonal evolution or acquired resistance during anti-cancer treatment. Of the 15 patients with 33 novel mutations at PD, 10 had 14 pathogenic mutations in 12 genes (Table 2). We visualized these pathogenic mutations in addition to other mutations (Fig. 2). The VAF values across different sample types were visualized in 8 patients who acquired actionable SNVs or small indels at PD (Fig. 2A). Among these, 6 patients (13.0%) acquired 9 loss-of-function mutations at PD: (a) *APC* S2223* in GC025, (b) *TP53* R273P in GC050, (c) *CDH1* N297_Y302del in GC070, (d) *ARID1A* P146Qfs*86, *RHOA* K187Nfs*169, *TP53* R175H, and *RNF43* R519* in GC085, (e) *BRCA2* R324* in GC116, and (f) *APC* S1756_A1759dup in GC177. In the other two patients, two gain-of-function mutations emerged at PD: *PIK3CA* R88Q in GC058 and *CTNNB1* S37C in GC099. In contrast, the CNs of some oncogenes substantially increased in PD, resulting in gain-of-function mutations in three patients (Fig. 2B): *MYC* amplification (CN = 7.68) in GC047, *CCND1* amplification (CN = 7.82) in GC081, and *EGFR* amplification (CN = 14.78) in GC116.

**Table 2:** Newly emerged pathogenic somatic variants in PD-cfDNA samples

Patient	Gene	Classification	Nucleotide change	Amino acid change	VAF or CN at PD
GC025	*APC*	Stop gained	c.6668C > G	S2223*	1.05%
GC047	*MYC*	Amplification	–	–	7.68
GC050	*TP53*	Pathogenic* missense variant	c.818G > C	R273P	0.75%
GC058	*PIK3CA*	Pathogenic* missense variant	c.263G > A	R88Q	0.34%
GC070	*CDH1*	Inframe deletion	c.890_907del	N297_Y302del	0.54%
GC081	*CCND1*	Amplification	–	–	7.82
GC085	*ARID1A*	Frameshift variant	c.437del	P146Qfs*86	13.4%
	*RHOA*	Frameshift variant	c.561del	K187Nfs*169	2.44%
	*TP53*	Pathogenic* missense variant	c.524G > A	R175H	0.61%
	*RNF43*	Stop gained	c.1555C > T	R519*	1.25%
GC099	*CTNNB1*	Pathogenic* missense variant	c.110C > G	S37C	0.55%
GC116	*BRCA2*	Stop gained	c.970A > T	R324*	0.83%
	*EGFR*	Amplification	–	–	14.78
GC177	*APC*	Inframe insertion	c.5267_5268insGTCTTCTGCGTC	S1756_A1759dup	0.49%

*According to ClinVar

*VAF* variant allele frequency, *CN* copy number, *PD* progressive disease, *cfDNA* cell-free DNA

### Survival outcomes according to DNA concentration and VAF

**Fig. 3:**
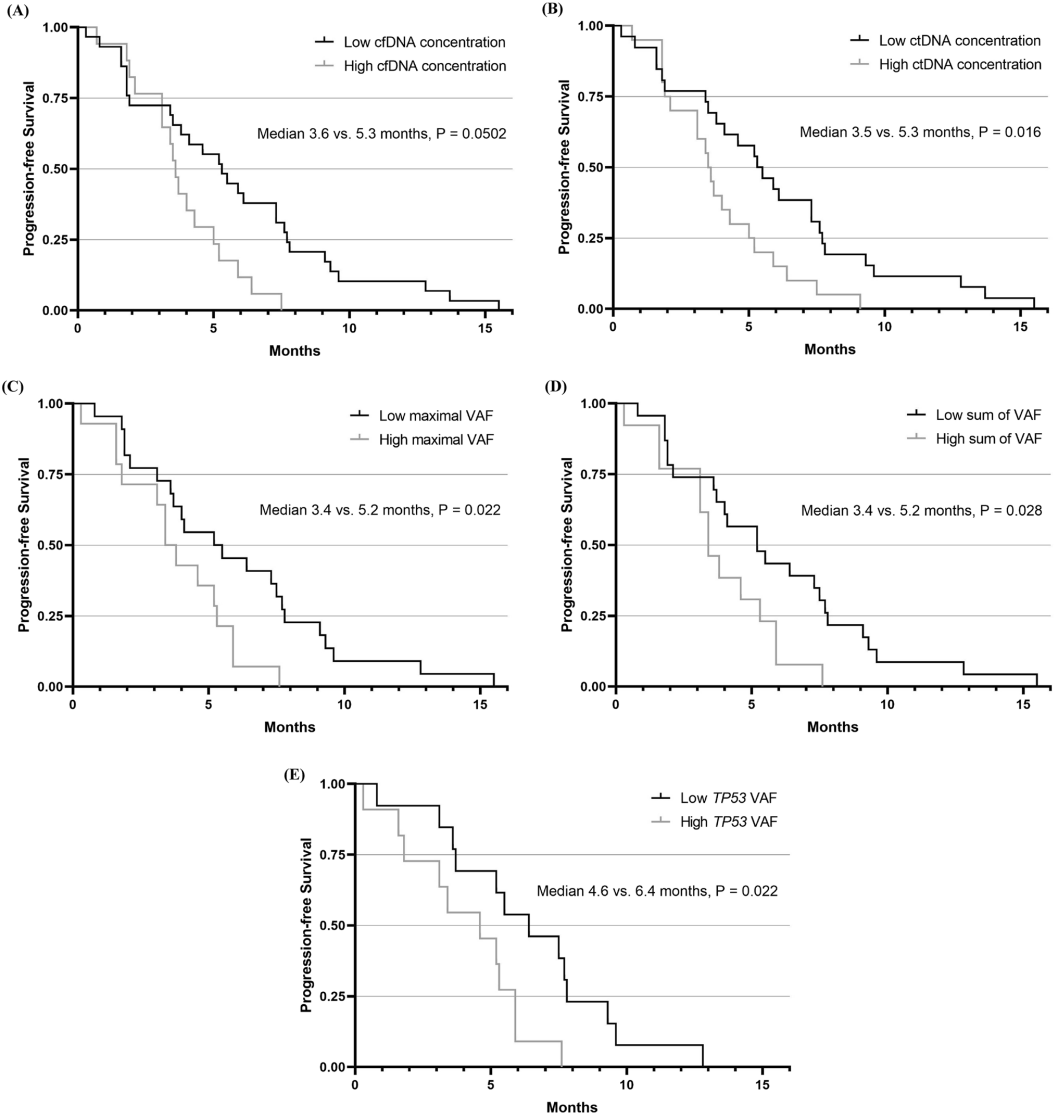
PFS according to DNA concentration and VAF. **A** Patients with a high cfDNA concentration (> 17.27 ng/µL) tended to have shorter PFS (*P* = 0.0502). **B** Patients with a high ctDNA concentration (> 4.38 ng/µL) at 110–160 bp had significantly shorter PFS (*P* = 0.016). **C** Among patients with any mutations in the baseline-cfDNA (*N* = 36), those with higher maximal VAF values (> 0.1045) demonstrated significantly worse PFS (*P* = 0.022). **D** Patients with a higher sum of VAF values (> 0.2071) had significantly shorter PFS (*P* = 0.028). **E** Among patients harboring *TP53* mutations (*N* = 24), those with high *TP53* VAF values (> 0.1014) showed significantly worse PFS (*P* = 0.022). *PD* progressive disease, *cfDNA* cell-free DNA, *PFS* progression-free survival

The median cfDNA concentration of the baseline-cfDNA samples was 12.94 (range, 3.44–141.40) ng/µL. Next, we calculated the concentration of the circulating tumor DNA (ctDNA) fraction at 110–160 bp based on previous literature [15]. The median concentration of ctDNA at the baseline was 3.91 (range, 0.81–46.63) ng/µL. Patients with a high cfDNA concentration greater than 17.27 ng/µL tended to have a shorter PFS (median 3.6 [95% CI, 3.2–4.0] vs. 5.3 [95% CI, 3.7–6.9] months, P = 0.0502, Fig. 3A) and OS (median 6.6 [95% CI, 4.2–9.0] vs. 11.1 [95% CI, 8.8–13.4] months, P = 0.123, Supplementary Fig. 3A). Patients with a high ctDNA concentration in the 110–160 bp range, with greater than 4.38 ng/µL had a significantly shorter PFS (median 3.5 [95% CI, 3.1–3.9] vs. 5.3 [95% CI, 3.7–6.9] months, P = 0.016, Fig. 3B) with a tendency for shorter OS (median 6.6 [95% CI, 3.3–9.9] vs. 11.1 [95% CI, 8.7–13.5] months, P = 0.139, Supplementary Fig. 3B).

In patients harboring mutations in the baseline-cfDNA (N = 36), the median maximal VAF was 0.0669 (range, 0.0017–0.6351). Patients with higher maximal VAF values greater than 0.1045 showed significantly shorter PFS (median 3.4 [95% CI, 2.5–4.3] vs. 5.2 [95% CI, 2.4–8.0] months, P = 0.022, Fig. 3C) with a tendency for shorter OS (median 6.2 [95% CI, 3.8–8.6] vs. 11.1 [95% CI, 8.7–13.5] months, P = 0.132, Supplementary Fig. 3C). In this group (N = 36), patients with the sum of VAF values greater than 0.2071 demonstrated significantly shorter PFS (median 3.4 [95% CI, 2.6–4.2] vs. 5.2 [95% CI, 3.0–7.4] months, P = 0.028, Fig. 3D) with a tendency for shorter OS (median 5.8 [95% CI, 3.3–8.3] vs. 11.1 [95% CI, 8.8–13.4] months, P = 0.181, Supplementary Fig. 3D). In patients with *TP53* mutations (N = 24), the median VAF values of *TP53* mutations were 0.0881 (range, 0.0017–0.6351). The PFS was significantly worse in those with high *TP53* VAF values greater than 0.1014 (median 4.6 [95% CI, 2.3–6.9] vs. 6.4 [95% CI, 3.7–9.1] months, P = 0.022, Fig. 3E) with a tendency for worse OS (median 7.8 [95% CI, 4.8–10.8] vs. 9.7 [95% CI, 5.4–14.0] months, P = 0.810, Supplementary Fig. 3E).

Univariable analysis was performed on the association between clinical characteristics or cfDNA-related factors and PFS (Supplementary Table 3). Because there was no significant variable related to clinical characteristics in the univariable analysis for PFS, we conducted multivariable analyses by adjusting age and sex. As a result, a high ctDNA concentration (> 4.38 ng/µL) at 110–160 bp was still statistically significant (hazard ratio [HR] 2.16 [95% CI, 1.14–4.11], P = 0.018) (Supplementary Table 4). However, high maximal VAF values (HR 2.10 [95% CI, 0.91–4.82], P = 0.082), a high sum of VAF values (HR 2.10 [95% CI, 0.95–4.66], P = 0.067), and high *TP53* VAF values (HR 2.74 [95% CI, 0.94–7.98], P = 0.065) could not maintain statistical significance due in part to the small sample size.

## Discussion

This study compared cancer panel data obtained using ttDNA with baseline- and PD-cfDNA data from 46 patients with mGC who underwent palliative second-line paclitaxel plus ramucirumab treatment. Our cfDNA platform detected somatic mutations better than conventional ttDNA-based cancer panels. Compared with ttDNA testing alone, 32 patients (70.0%) benefited from a single baseline-cfDNA test for detecting somatic variants. The number of patients increased to 34 (73.9%) when both baseline- and PD-cfDNA were analyzed. Further, for the top four genes, our cfDNA analysis had a sensitivity of 71.8% for detecting variants found in ttDNA. However, our results suggest that cfDNA analysis cannot completely replace conventional ttDNA-based cancer panels because some variants that could have been identified using conventional ttDNA analysis may still be missed by cfDNA. However, cfDNA analysis could detect additional mutations that could have been missed by ttDNA analysis alone.

Moreover, the advantage of cfDNA is that it allows for repeated testing even when a tumor biopsy is not feasible. Notably, cfDNA analysis enables easy identification of somatic mutations that explain acquired resistance, such as *EGFR* T790M in lung adenocarcinoma [16]. Previous studies on mGC have utilized serial cfDNA monitoring in patients treated with *HER2*-targeted agents [17–21] or immune checkpoint inhibitors [22, 23]. In those receiving the *HER2* antibody–drug conjugate trastuzumab deruxtecan, *MET*, *EGFR*, and *FGFR2* amplifications and *PIK3CA* mutations were associated with lower response rates whereas *HER2* mutations tended to be associated with higher response rates [21]. In those treated with immune checkpoint inhibitors such as nivolumab and pembrolizumab, high tumor mutational burden at baseline and VAF change during treatment predicted response [22, 23]. However, to the best of our knowledge, no studies have been conducted on patients receiving paclitaxel plus ramucirumab. In our study, compared with both ttDNA and baseline-cfDNA, repetitive cfDNA testing at PD identified 14 newly emerged pathogenic variants in 10 patients (21.7%), including nine loss-of-function and five gain-of-function variants. While no clear correlation was found between these variants and acquired resistance to paclitaxel or ramucirumab, five patients with gain-of-function mutations, including *PIK3CA* R88Q, *CTNNB1* S37C, and *MYC*, *CCND1*, and *EGFR* amplifications, could be potential candidates for clinical trials [24–28].

In TCGA data of gastric adenocarcinoma [5], the prevalence of *TP53*, *CDH1*, *KRAS*, and *APC* mutations including amplification and deep deletion was 48%, 11%, 16%, and 18%, which is different from our data of 58.7%, 26.1%, 21.7%, and 13.0%. The difference between TCGA data and ours would be mainly attributed to the different sensitivity of the two detection methods. TCGA data were generated by whole exome sequencing (WES) with a mean coverage depth of ~ 120 × , less than 1/10 of our cfDNA platform. Therefore, our data suggests that our cfDNA platform was comparable to WES using ttDNA in terms of variant detection performance. In TCGA data, mutations may not have been well detected by WES in samples with low tumor purity. In the cfDNA platform, deep deletion may not be detected better than WES because of limited information on tumor purity and ploidy in cfDNA samples. Other contributing factors may include differences in ethnicity (Asian vs. non-Asian) and stage (IV vs. I–III) between the two cohorts.

There is no proven biomarker for predicting the response to anti-angiogenic agents, including ramucirumab. Our study demonstrated that the ctDNA fraction concentration at 110–160 bp and VAF values were associated with longer PFS in our cohort. Previous studies have suggested that cfDNA concentration at the baseline predicts the prognosis of patients with metastatic cancers [29–31]. However, the predictive and prognostic roles of ctDNA fraction concentrations at 110–160 bp have not been previously reported. In our cohort, patients with high cfDNA concentrations tended to have shorter PFS, with borderline significance. Thus, we hypothesized that the 110–160 bp fraction concentration of cfDNA may better predict PFS because ctDNA is enriched in this fraction [15]. Patients with a high 110–160 bp fraction concentration had a significantly shorter PFS. Our results may not be sufficient to determine whether this finding is specific to the ramucirumab plus paclitaxel regimen. Therefore, additional validation studies with enough sample sizes are warranted.

Available lines of evidence suggest that baseline-cfDNA VAF values may reflect the overall tumor burden [32, 33]. In our study, high maximal VAF (P = 0.022), high sum of VAF (P = 0.028), and high *TP53* VAF (P = 0.022) were significantly associated with worse PFS in the univariable analysis, which is in line with previous data from patients with gastroesophageal adenocarcinoma [34]. Accurate estimation of the overall tumor burden in patients using conventional imaging techniques is challenging. In contrast, baseline-cfDNA analysis is a relatively simple technique for estimating the overall tumor burden. However, even with constant tumor purity, VAF values can be significantly affected by the loss-of-heterozygosity of a specific locus [35]. Therefore, combining VAF with loss-of-heterozygosity can provide a more comprehensive analysis.

A limitation of this study is that the ttDNA dataset was obtained from four different cancer panels. Thus, some genes in our cfDNA platform were not covered in the four ttDNA-based cancer panels, as indicated by the X-mark in Fig. 1. Nevertheless, to clarify the variant detection performance of the four panels, we reported their performance when our cfDNA platform was used as the gold standard. In addition, we could not analyze the effects of resistant clones in the PD-cfDNA data on patient survival because the driver mutations of the resistant clones were diverse and we could not estimate the time points of the resistant clone emergence using the PD-cfDNA data.

Overall, our cfDNA platform demonstrated considerable performance in detecting somatic mutations compared with conventional ttDNA-based cancer panels, identifying additional somatic mutations that were otherwise missed by ttDNA alone. Moreover, PD-cfDNA analysis revealed novel pathogenic mutations that developed during treatment, implicating the clonal evolution of cancer or acquired resistance during anti-cancer treatment. In addition, the ctDNA fraction concentration at 110–160 bp and the VAF values of the baseline-cfDNA samples were associated with PFS in patients receiving paclitaxel plus ramucirumab treatment.

## Supplementary Information

Below is the link to the electronic supplementary material.

Supplementary file1 (PPTX 402 KB)

Supplementary file2 (DOCX 26 KB)
