# Supplementary material for: Pairwise analysis of plasma cell-free DNA before and after palliative second-line paclitaxel plus ramucirumab treatment in patients with metastatic gastric cancer
Source: Gastric Cancer. 2025 Mar 27;28(4):620–30. doi: 10.1007/s10120-025-01604-y (PMC12174237; doi:10.1007/s10120-025-01604-y)
Supplement: Supplementary file 1 — Supplementary file1 (PPTX 402 KB) [file 10120_2025_1604_MOESM1_ESM.pptx]

## Slide 1
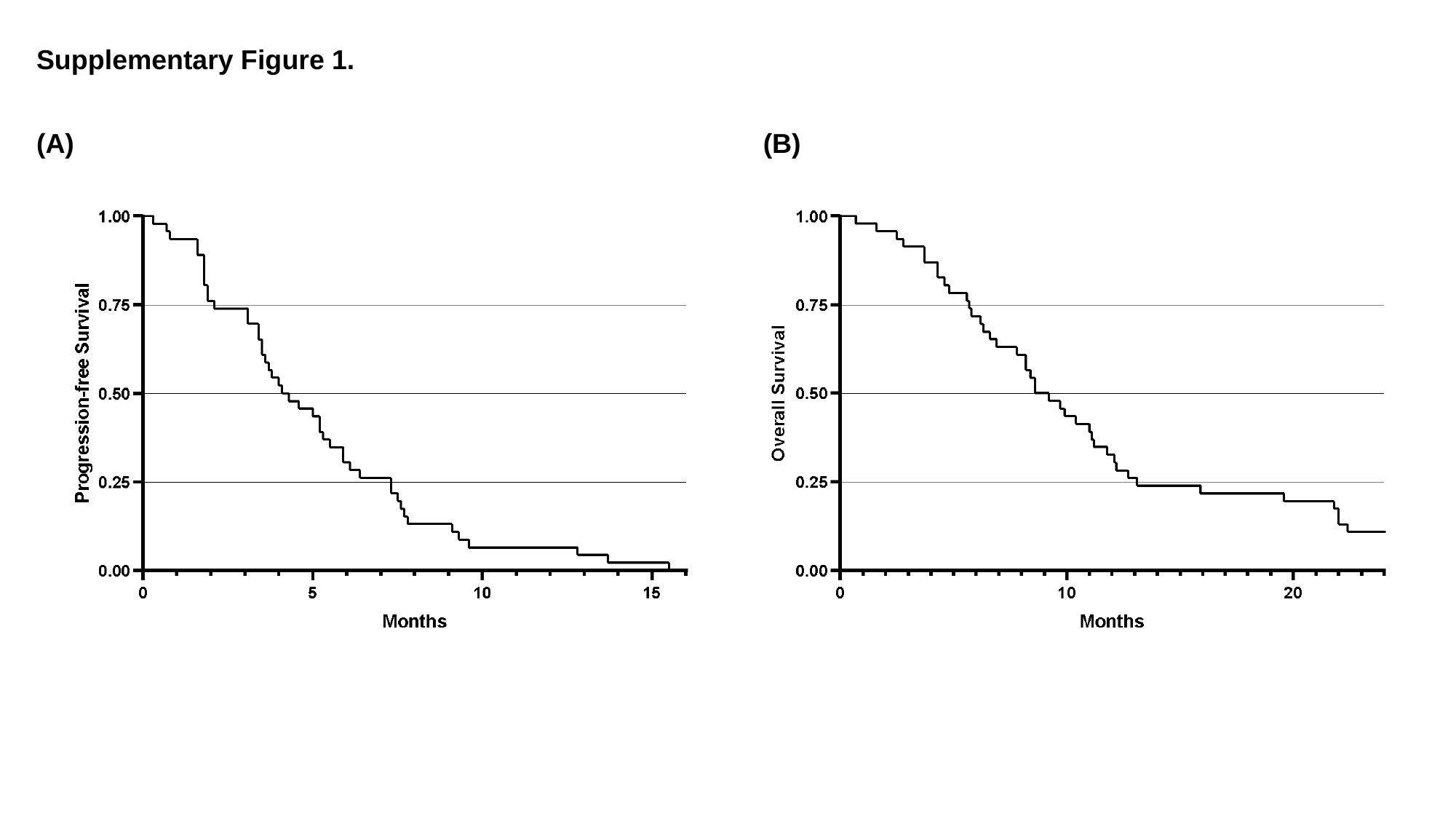

Supplementary Figure 1.
(A)
(B)

## Slide 2
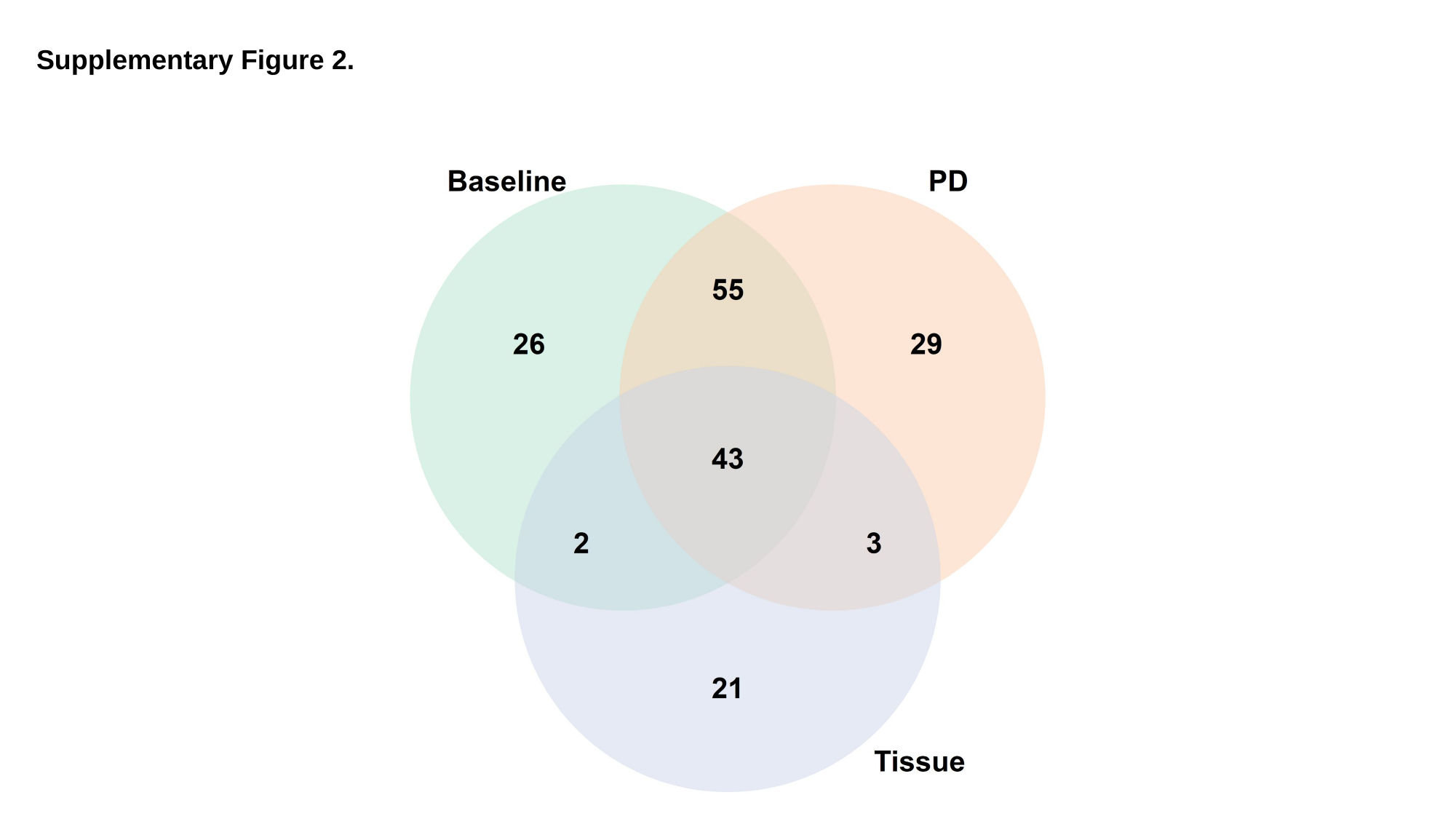

Supplementary Figure 2.

## Slide 3
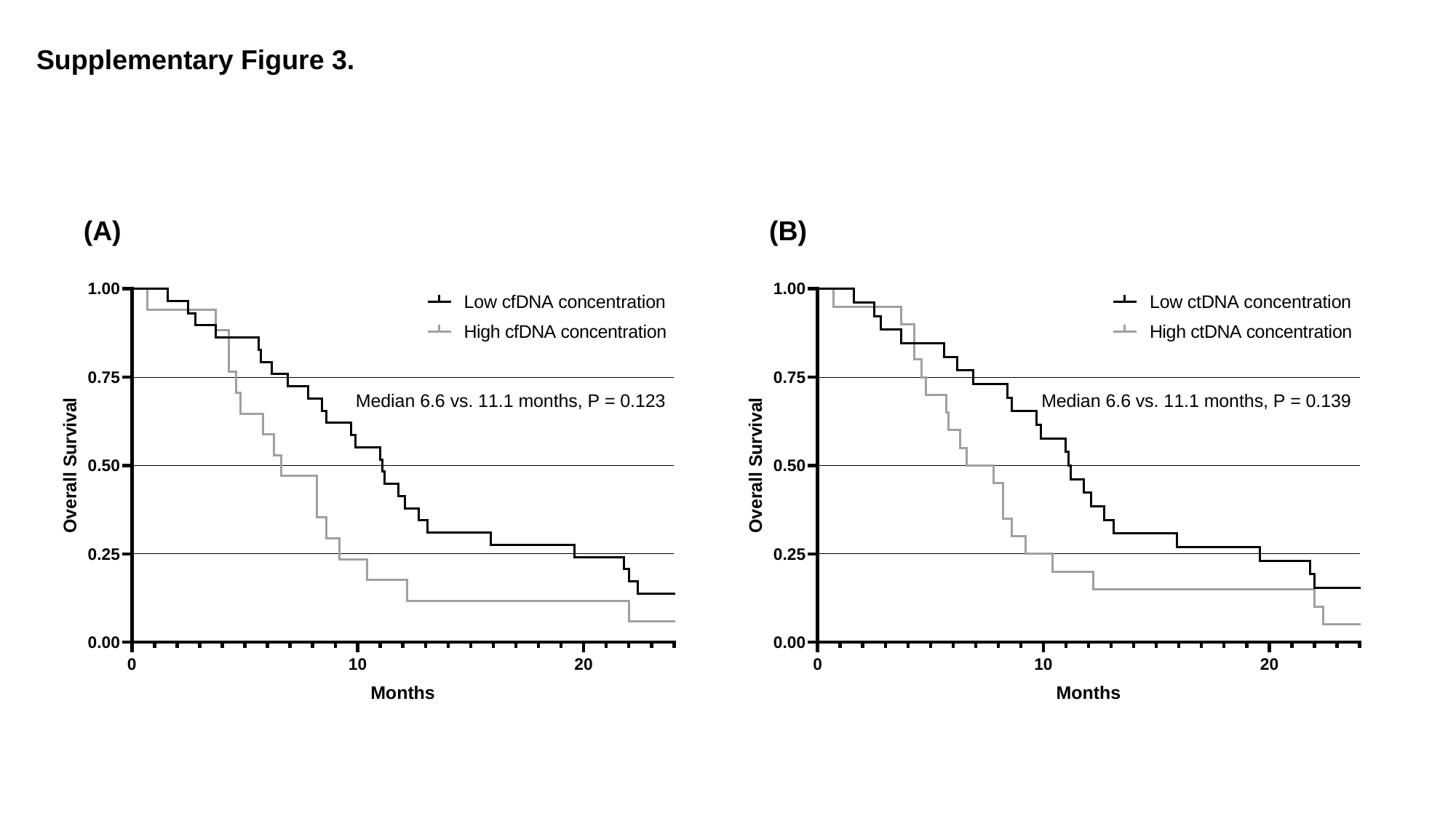

Supplementary Figure 3.
(A)
(B)

## Slide 4
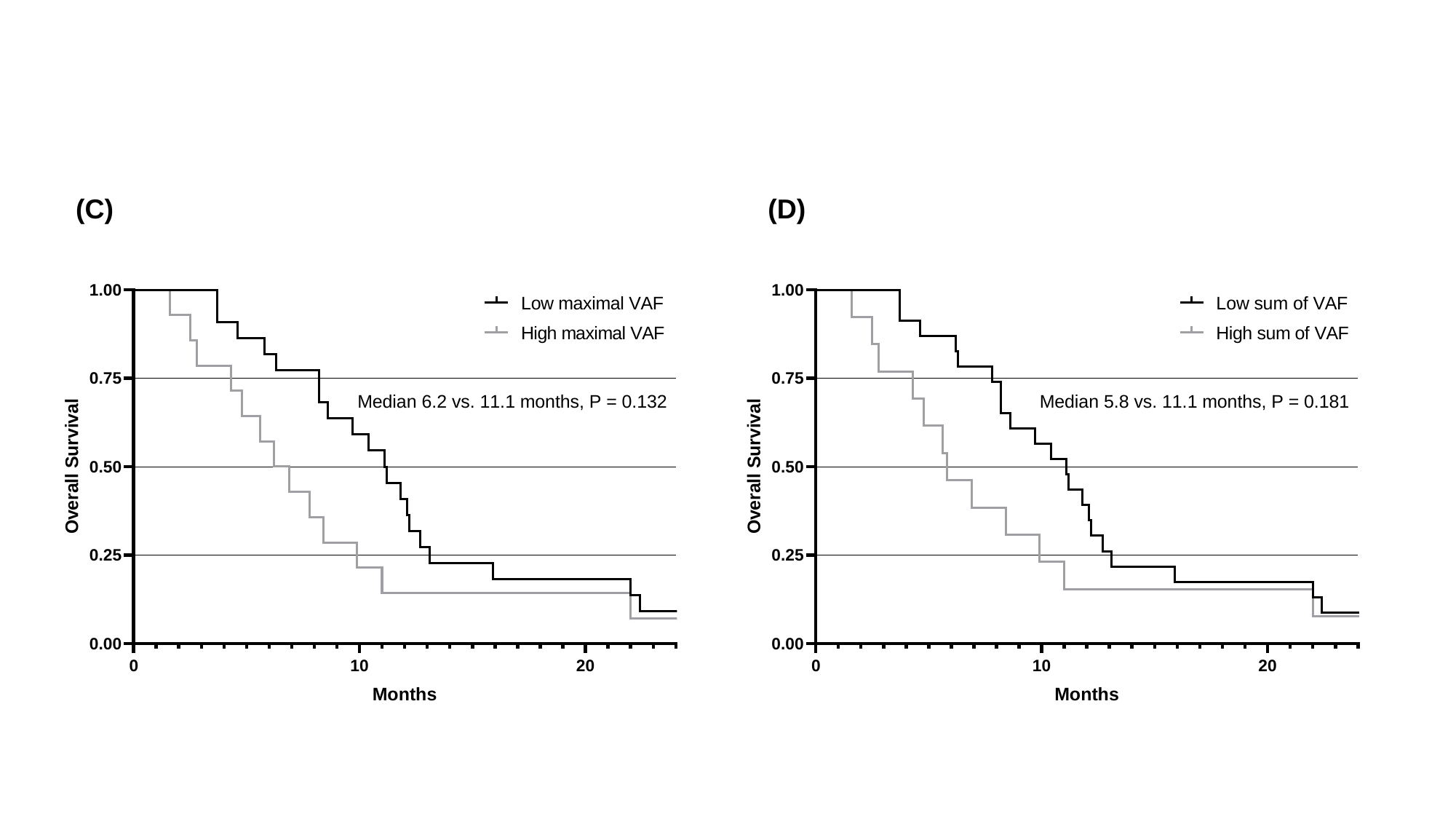

(C)
(D)

## Slide 5
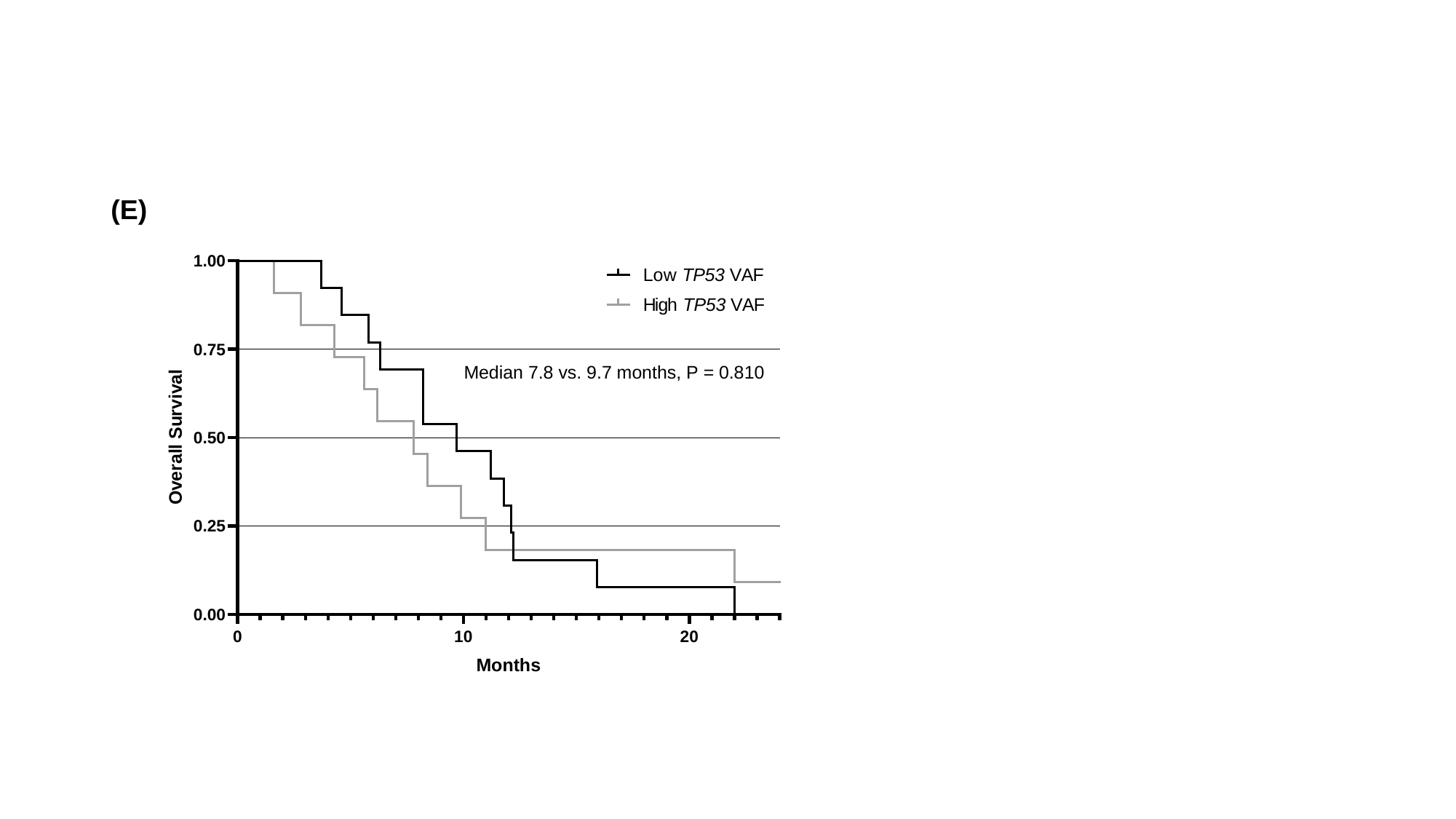

(E)
