# Supplementary material for: Pairwise analysis of plasma cell-free DNA before and after palliative second-line paclitaxel plus ramucirumab treatment in patients with metastatic gastric cancer
Source: Gastric Cancer. 2025 Mar 27;28(4):620–30. doi: 10.1007/s10120-025-01604-y (PMC12174237; doi:10.1007/s10120-025-01604-y)
Supplement: Supplementary file 2 — Supplementary file2 (DOCX 26 KB) [file 10120_2025_1604_MOESM2_ESM.docx]

# **Supplementary Figures**

**Supplementary Figure 1. Kaplan**–**Meier survival analysis.**

(A) The median PFS of patients after palliative second-line treatment was 4.1 (95% CI, 2.7–5.5) months. (B) Their median OS was 8.6 (95% CI, 6.7–10.5) months.

Abbreviations: PFS = progression-free survival; CI = confidence interval; OS = overall survival.

**Supplementary Figure 2. Venn diagram of detected mutations according to sample type.**

Among 179 mutations, PD-cfDNA (N =130) showed the highest occurrence, followed by baseline-cfDNA (N =126) and ttDNA (N =69).

Abbreviations: PD = progressive disease; cfDNA = cell-free DNA; ttDNA = tumor tissue DNA.

**Supplementary Figure 3. OS according to DNA concentration and VAF.**

(A) Patients with a high cfDNA concentration (> 17.27 ng/µL) tended to have shorter OS (P = 0.123). (B) Patients with a high ctDNA concentration (> 4.38 ng/µL) at 110–160 bp tended to have shorter OS (P = 0.139). (C) In patients harboring any mutations in the baseline-cfDNA (N = 36), those with higher maximal VAF values (> 0.1045) tended to have shorter OS (P = 0.132). (D) Patients with a higher sum of VAF values (> 0.2071) tended to have shorter OS (P = 0.181). (E) Among patients with *TP53* mutations (N = 24), the OS tended to be worse in those with high *TP53* VAF (> 0.1014) (P = 0.810).

Abbreviations: OS = overall survival; VAF = variant allele frequency; cfDNA = cell-free DNA; ctDNA = circulating tumor DNA.

# **Supplementary Tables**

**Supplementary Table 1. Comparison of targeted panels.**

Abbreviations: cfDNA = cell-free DNA; ttDNA = tumor tissue DNA; AL100 = AlphaLiquid®100; SNUBH_V1 = SNUBH Pan-Cancer Panel version 1; SNUBH_V2 = SNUBH Pan-Cancer Panel version 2; TS500 = Illumina TruSight Oncology 500.

**Supplementary Table 2. Tumor response outcomes of palliative second-line treatment in 34 patients with measurable lesions based on RECIST 1.1.**

| **Best response** | **n (%)** |
| --- | --- |
| **PR**  **SD**  **PD** | 8 (23.5)  16 (47.1)  10 (29.4) |

Abbreviations: RECIST = Response Evaluation Criteria in Solid Tumors; PR = partial response; SD = stable disease; PD = progressive disease.

**Supplementary Table 3. Univariable analysis for PFS.**

| **Clinical characteristics** | | **No. of patients** | **Median PFS (95% CI)** | **P-value** |
| --- | --- | --- | --- | --- |
| **Age** | **< 65 years**  **≥ 65 years** | 35 (76.1)  11 (23.9) | 4.0 (2.4–5.6)  4.6 (0.2–9.0) | 0.272 |
| **Sex** | **Male**  **Female** | 27 (58.7)  19 (41.3) | 3.8 (3.1–4.5)  5.2 (3.5–6.9) | 0.146 |
| **Peritoneal metastasis** | **Present**  **Absent** | 32 (69.6)  14 (30.4) | 4.3 (2.9–5.7)  3.4 (2.9–3.9) | 0.931 |
| **Liver metastasis** | **Present**  **Absent** | 16 (34.8)  30 (65.2) | 3.4 (0.5–6.3)  4.6 (3.1–6.1) | 0.104 |
| **No. of involved organs** | **< 3**  **≥ 3** | 27 (58.7)  19 (41.3) | 4.6 (2.9–6.3)  3.8 (2.5–5.1) | 0.882 |
| **Histologic type^*^** | **TA**  **PCC**  **Other** | 21 (45.7)  16 (34.8)  9 (19.6) | 4.3 (1.8–6.8)  3.7 (0.8–6.6)  4.1 (3.8–4.4) | 0.604 |
| **Differentiation** | **W-M/D**  **P/D**  **Unknown** | 14 (30.4)  18 (39.1)  14 (30.4) | 4.0 (0.9–7.1)  3.7 (2.9–4.5)  5.0 (3.3–6.7) | 0.170 |
| **Lauren classification** | **Intestinal**  **Diffuse**  **Others** | 10 (21.7)  22 (47.8)  14 (30.4) | 3.4 (0.9–5.9)  4.0 (2.4–5.6)  4.6 (2.9–6.3) | 0.723 |
| ***HER2*** | **Positive**  **Negative** | 6 (13.0)  40 (87.0) | 1.8 (0.0–6.0)  4.1 (2.2–6.0) | 0.448 |
| **Disease status at palliative treatment** | **Recurrent**  **Initially metastatic** | 14 (30.4)  32 (69.6) | 3.5 (0.9–6.1)  4.6 (3.0–6.2) | 0.942 |
| **Measurable lesion by RECIST 1.1** | **Measurable**  **Non-measurable** | 34 (73.9)  12 (26.1) | 4.1 (2.4–5.8)  3.7 (2.5–4.9) | 0.738 |

Abbreviations: PFS = progression-free survival; CI = confidence interval; TA = tubular adenocarcinoma; PCC = poorly cohesive carcinoma; W-M/D = well-to-moderate differentiation; P/D = poor differentiation; RECIST = Response Evaluation Criteria in Solid Tumors.

^*^Histological type according to the WHO 2010 classification.

**Supplementary Table 4. Multivariable analysis for PFS.**

| **Clinical characteristics** | | **No. of patients** | **Hazard ratio (95% CI)** | **P-value** |
| --- | --- | --- | --- | --- |
| **Age** | **< 65 years**  **≥ 65 years** | 35 (76.1)  11 (23.9) | Reference  0.51 (0.24–1.10) | 0.085 |
| **Sex** | **Male**  **Female** | 27 (58.7)  19 (41.3) | Reference  0.52 (0.27–1.01) | 0.054 |
| **ctDNA concentration at 110–160 bp** | **< 4.38 ng/µL**  **≥ 4.38 ng/µL** | 26 (56.5)  20 (43.5) | Reference  2.16 (1.14–4.11) | 0.018 |

Abbreviations: CI = confidence interval; ctDNA = circulating tumor DNA; bp = base pair.
